# Preoperative Radar Localisation of Impalpable Breast and Axillary Lesions: Initial Experience in an Australian Tertiary Centre

**DOI:** 10.1111/1754-9485.70073

**Published:** 2026-02-12

**Authors:** Grace Carpenter, Coralie Simoni, Lucy Lu, Jacqueline Eskander, Tracy Liu, Jennifer Yuill, Sophie Nightingale, Margaret Nguyen, Joseph Paiva

**Affiliations:** ^1^ Department of Medical Imaging Western Health Melbourne Victoria Australia; ^2^ Department of Breast Surgery Western Health Melbourne Victoria Australia

**Keywords:** axillary surgery, breast conserving surgery, electromagnetic localisation, image‐guided localisation, wire‐free localisation

## Abstract

**Introduction:**

Image‐guided radar localisation (RL) is a wire‐free alternative to hookwire localisation (HWL) for preoperative localisation of impalpable breast and axillary lesions, with potential logistical and technical advantages. This study assesses clinical utility, accuracy of placement and surgical oncologic outcome metrics for RL using the SCOUT reflector compared to HWL after introduction in a tertiary Australian breast service.

**Methods:**

Retrospective comparative cohort analysis of consecutive RL with a contemporaneous historical HWL cohort of similar size (between January 2022 and March 2025) was performed. Key outcome markers included device migration, localisation procedure duration, retrieval issues, surgical margin status and breast malignancy re‐excision rates.

**Results:**

287 patients were included (RL *n* = 144; HWL *n* = 143). No significant device migration was seen with RL compared to HWL (0.0% vs. 4.2%; *p* = 0.04) and retrieval issues were less common (4.2% vs. 9.8%; *p* = 0.10). Excluding MRI placement, procedure duration was shorter with RL (23 min vs. 28 min; *p* < 0.01). Negative, close, and positive margins were similar and re‐excision rates for breast malignancy comparable (12.5% RL vs 16.8% HWL, *p* = 0.42).

**Conclusion:**

RL is a safe, accurate localisation technique, demonstrating significantly fewer migration issues and fewer retrieval issues than HWL, while maintaining equivalent surgical oncologic outcomes. It offers logistical benefits of placement at any time prior to surgery (including time‐of‐biopsy placement), optimisation of radiology and surgical workflows, and versatility of axillary node and MRI‐guided placement and compatibility.

## 1 Introduction

Breast cancer is the most common cancer affecting Australian women, with 20,973 women diagnosed with breast cancer in 2024 [1]. Breast cancer outcomes have significantly improved recently due to several factors, including population‐based screening mammography, resulting in increased detection of early impalpable breast cancers and advances in targeted therapies and surgical techniques. Neoadjuvant treatment, particularly chemotherapy, is increasingly offered to certain patients, downstaging many cancers prior to surgery, rendering them impalpable [2]. Many of these cases are amenable to breast conserving surgery (BCS) followed by adjuvant radiotherapy, with studies showing similar long‐term survival rates to mastectomy [3, 4].

Historically, the gold standard management of node‐positive breast cancer has been axillary lymph node dissection (ALND) to complete axillary staging. Due to the inherent morbidity associated with ALND, notably arm lymphoedema, there has been a paradigm shift towards de‐escalating axillary surgery. This has led to the development of targeted axillary dissections (TAD), where positive nodes at the time of diagnosis are marked with a clip and removed after neoadjuvant chemotherapy, along with sentinel nodes, providing less invasive axillary staging [5, 6].

For impalpable breast cancers and TAD, accurate preoperative localisation is critical for successful surgical excision. The most widely used and established technique, traditional hookwire localisation (HWL), has well‐documented limitations. These include scheduling constraints due to coordination of same‐day hookwire insertion and surgery; risk of wire breakage, migration or dislodgement; discrepancy in hookwire entry site/tract and preferred surgical approach; technically difficult insertion for axillary nodes; and patient discomfort [7]. These challenges impact workflow efficiency, surgical precision, and patient experience.

Consequently, several non‐wire localisation technologies have emerged [8], including carbon tracking, radioactive iodine seeds (ROLLIS), magnetic seeds (Magseed [9, 10, 11], Pintuition), and radiofrequency identification (RFID) tags [12]. Each aims to improve surgical and radiology logistics (by decoupling the time of image‐guided insertion and surgery), patient comfort, and accuracy. Each has limitations, including restricting the surgical incision path to carbon track for carbon tracking; logistical and regulatory challenges around radioactive material/specimens for radioactive seeds; MRI artefact with magnetic seeds, precluding follow‐up MRI imaging; and lack of MRI compatibility of insertion systems for some aforementioned technologies [7].

**FIGURE 1:**
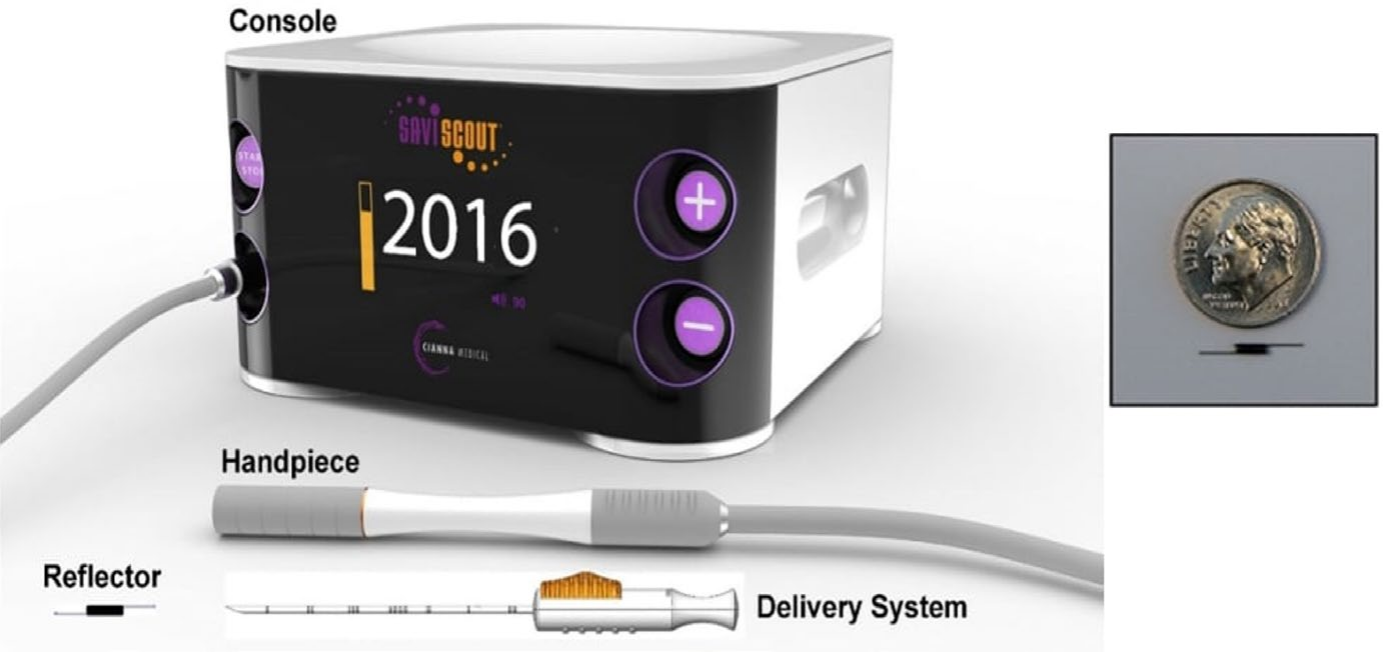
SCOUT radar localisation device delivery system and console. Image courtesy of Merit Medical Systems.

SCOUT radar localisation (RL; Merit Medical Systems, South Jordan, UT, USA) represents another step forward, offering non‐radioactive and non‐magnetic wireless lesion marking. A 12 mm reflector is placed into the target lesion using a 16 gauge introducer needle, utilising ultrasound, mammographic or MRI guidance. During surgery, the reflector is activated by infrared light from a console probe and reflects back electromagnetic wave signals (see Figure 1). This provides real‐time proximity information, enabling precise surgical guidance within 1 mm accuracy and 360° detection. A smaller 8 mm ‘mini‐reflector’ is utilised for axillary lymph node placement.

SCOUT is inert, has ‘permanent implant’ status, and can be placed any time before surgery, supporting flexible scheduling and decoupling radiology and surgery services. Unlike other wireless alternatives, SCOUT can be inserted under MRI guidance with minimal MRI artefact in literature to date [13, 14]. SCOUT is suitable for localising breast lesions and axillary nodes, potentially increasing clinical versatility. The RL device is located entirely within the patient, with no tract to follow for surgical dissection, allowing flexibility in surgical approach to optimise oncoplastic results.

This study evaluates the performance of SCOUT radar localisation in an Australian tertiary centre. A similar sized cohort of HWL cases provides comparative context.

## 2 Methods

### 2.1 Study Design and Setting

We conducted a single‐centre retrospective cohort study at a high‐volume tertiary breast unit within Sunshine Hospital, Western Health, Victoria, Australia. Ethics approval was granted by the Western Health Low Risk Ethics Committee (LR/25/WH/116869) with waiver of consent for use of de‐identified data.

### 2.2 Participants

SCOUT was introduced at our institution in September 2023. Between September 2023 and March 2025, 151 consecutive patients who underwent RL for impalpable breast lesions or axillary lymph nodes for surgical excision (BCS or TAD) were included. A total of three RL patients were excluded: one due to having surgery at a different institution, one to avoid confounding factors due to having both RL and HWL used for bracketing, and one axillary RL due to histopathological diagnosis of lymphoma. Four additional patients were excluded due to surgery still pending at time of analysis.

The most recent cohort of 147 patients who underwent HWL for similar indications was included for comparison; these patients underwent treatment between January 2022 and December 2024. Two additional patients were excluded due to missing surgical data, one patient was excluded due to being the only computed tomography‐guided RL, and one patient was excluded due to both RL and HWL being used for bracketing.

Final numbers after exclusions were 144 RL patients and 143 HWL patients.

### 2.3 Localisation Techniques and Definitions

For the RL cohort, reflectors were deployed under ultrasound, mammographic, or MRI guidance (depending on optimal modality for lesion visibility) using a standard applicator device after local anaesthesia. Reflectors were placed either at the time of biopsy for unifocal BI‐RADS 5 (high risk) lesions, which require excision regardless of histology, or in a separate preoperative localisation appointment. For the HWL cohort, a stainless steel 0.3 mm diameter Kopans hookwire was inserted in all cases on the day of surgery under ultrasound or mammographic guidance, after local anaesthesia. All localisations (RL and HWL) were performed by specialist breast radiologists or their supervised trainee registrars, in a tertiary public teaching hospital.

### 2.4 Outcome Markers

The total duration of the localisation intervention was collected by the first and last image saved on the Picture Archiving and Communications System (PACS) for the modality used, to glean an objective measurement of insertion time of the localisation device (RL or hookwire), excluding periprocedural workflow issues such as unexpected delays related to patient/nurse/radiographer/radiologist pre‐procedure. If more than one SCOUT or hookwire was inserted during a single procedure, the total duration of the procedure was divided by the number of localisation devices inserted at the time.

Presence of haematoma at time of localisation and localiser migration data were collected. Migration was assessed on mammogram post localisation and defined as distance between biopsy clip (or lesion if no biopsy clip) and hookwire, or the closest antenna of SCOUT. Migration was defined as > 10 mm displacement from target biopsy clip/lesion, as per accepted definition in literature [15]. Localisation success was defined as successful intraoperative detection and retrieval of the RL reflector.

Surgical margin status and breast malignancy re‐excision rates were assessed as key outcome markers, as per standard surgical reporting. Margins at surgery were assessed using pathology report and divided into four categories: (1) negative, (2) close (within 2 mm for ductal carcinoma in situ [DCIS]), (3) positive, or (4) benign pathology. Margins were classified as negative for mastectomy, axillary dissection, and pathological complete response (pCR) after neoadjuvant therapy. Need for re‐excision and rates of re‐excision for breast malignancy in each group were analysed from patient records. All surgeries were performed by specialist breast surgeons or their supervised trainee registrars.

### 2.5 Data Collection and Statistical Analysis

Data was extracted from the electronic medical record (EMR), PACS and Pathology Database, and entered into a REDCap database by trained researchers. Descriptive statistics summarised patient, lesion and procedural characteristics. Group comparisons used Chi‐square tests or Fisher's exact tests for categorical variables and *t*‐tests or Mann–Whitney *U* tests for continuous variables after Shapiro–Wilk testing for normality. Significance was set at α = 0.05. Analyses were performed in the statistical computing system ‘R' [16].

## 3 Results

### 3.1 Study Cohort and Demographics

144 RL and 143 HWL patients met the inclusion criteria. Mean age was 57 in the HWL cohort and 58 in the RL cohort. All patients were female.

### 3.2 Localisation Characteristics

SCOUT was placed under ultrasound (64.6%), mammographic (31.9%) or MRI (3.5%) guidance, with 10.4% deployed at the time of biopsy in BI‐RADS 5 lesions. HWL was performed under ultrasound (67.1%) or mammographic (32.9%) guidance. There were more axillary RL than HWL (10.4% vs. 4.2%; *p* = 0.07). Localiser migration did not occur with RL (0.0%), significantly lower than HWL (4.2%; *p* = 0.04).

Haematoma at time of localisation from prior biopsy was recorded in 2.8% of RL cases and was not recorded for HWL. Multiple localisation devices were placed in the same patient in 22.9% of RL and 28.0% of HWL cases, for bracketing (5.6% in RL vs. 6.3% in HWL) or multiple lesions (17.4% in RL vs. 21.7% in HWL). RL was placed at time of biopsy (for BI‐RADS 5 lesions) in 15 patients (10.4%).

Table 1 summarises localisation characteristics and compares RL and HWL cohorts.

**TABLE 1:** Localisation characteristics.

Characteristic	All (*n* = 287)	HWL (*n* = 143)	RL (*n* = 144)
Imaging guidance modality, *n* (%)			
Ultrasound	189 (65.9)	96 (67.1)	93 (64.6)
Mammography	93 (32.4)	47 (32.9)	46 (31.9)
MRI	5 (1.7)	0 (0.0)	5 (3.5)
Lesion location, *n* (%)			
Breast	266 (92.7)	137 (95.8)	129 (89.6)
Axilla	21 (7.3)	6 (4.2)	15 (10.4)
Multiple localisers placed, *n* (%)			
Bracketing	17 (5.9)	9 (6.3)	8 (5.6)
Multiple lesions	56 (19.5)	31 (21.7)	25 (17.4)
Total	73 (25.4)	40 (28.0)	33 (22.9)
SCOUT placed at biopsy (BI‐RADS 5), *n* (%)	—	—	15 (10.4)
Complications, *n* (%)			
Localiser migration > 10 mm	6 (2.1)	6 (4.2)	0 (0.0)
Duration of localisation procedure (min), mean ± SD^a^	25 ± 13 (*n* = 282)	28 ± 14 (*n* = 143)	23 ± 12 (*n* = 139)

^a^ Excludes MRI‐guided procedures.

The duration of the device insertion procedure was shorter for RL than HWL when MRI placements were excluded (23 (±12) min vs. 28 (±14) min) (*p* < 0.01). Mammography‐guided placements had a mean duration of 23 min (±9) for RL and 27 (±15) min for HWL. Ultrasound‐guided placements had a mean duration of 23 (±13) minutes for RL and 28 (±14) minutes for HWL. MRI‐guided placements of RL had a mean duration of 38 (±8) minutes.

### 3.3 Surgical and Histopathology Outcomes

Please see Table 2 for a summary of surgical outcomes. In all cases (HWL and RL), the target lesions were retrieved successfully. Retrieval issues were less frequent with RL (6 RL (4.2%) vs 14 HWL (9.8%); *p* = 0.10) for combined breast and axillary lesions. With RL, 6 cases had retrieval issues at surgery, with 4 being secondary to large fatty breasts. In one case, the SCOUT malfunctioned at surgery, and intraoperative ultrasound was used for localisation. In another case, SCOUT was not found in the initial specimen, with further margins subsequently taken, which contained SCOUT. Hookwire retrieval issues were not related to large fatty breasts and were related to technical factors, e.g., multiple hookwires and dislodgement.

**TABLE 2:** Surgical breast outcomes (excluding axillary nodes).

Outcome	All (*n* = 266)	HWL (*n* = 137)	RL (*n* = 129)
Margins, *n* (%)			
Negative	139 (52.2)	69 (50.4)	70 (54.3)
Close (≤ 2 mm)	41 (15.4)	19 (13.8)	22 (17.1)
Positive	11 (4.1)	7 (5.1)	4 (3.1)
Benign pathology	75 (28.1)	42 (30.7)	33 (25.6)
Retrieval issues, *n* (%)	19 (7.1)	14 (10.2)	5 (3.9)
Re‐excision required for malignancy, *n* (%) excluding benign cases	28 (14.7)	16 (16.8)	12 (12.5)

Margin status was comparable (negative 54.3% vs. 50.4%; close 17.1% vs. 13.8%; positive 3.1% vs. 5.1%; benign 25.6% vs. 30.7%) and breast malignancy re‐excision rates were similar (12.5% vs. 16.8%) between RL and HWL groups, (*p* = 0.42). Pathological diagnoses (see Table 3) were relatively balanced across groups (benign 27.2%, DCIS only 19.2%, invasive carcinoma 43.5%), with a higher percentage of malignant histopathology overall. Localisations were performed for impalpable malignancies (most cases), or excisional biopsies for high‐risk or radiologic‐pathologic discordant lesions.

**TABLE 3:** Postoperative final histopathology results.

Diagnosis/Feature	All (*n* = 287)	HWL (*n* = 143)	RL (*n* = 144)
Histopathology, *n* (%)			
Benign	78 (27.2%)	45 (31.5%)	33 (22.9%)
DCIS only	55 (19.2%)	25 (17.5%)	30 (20.8%)
Invasive carcinoma	125 (43.5%)	65 (45.4%)	60 (41.7%)
Complete pathological response at surgery (no residual malignancy)	29 (10.1%)	8 (5.6%)	21 (14.6%)

## 4 Discussion

In this real‐world Australian cohort, RL demonstrated significantly lower device‐related complications than HWL, while preserving short‐term oncological outcomes.

### 4.1 Device‐Related Issues

Device migration occurred in 4.2% of HWL cases but did not occur in RL cases irrespective of the RL modality (*p* = 0.04), suggesting insertion is accurate across ultrasound, mammography and MRI, despite occasional presence of haematoma. This aligns with more recent international comparative analyses reporting a migration rate of < 1%, and suggests that the size of the reflector is a potential factor in mitigation of device dislodgement [13, 17]. The partially external nature of hookwires increases risk of migration (non‐existent with an internalised device), supported by our results. In our experience, RL is technically simple to insert. It is akin to insertion of a marker clip, utilising a simple applicator device, for ultrasound and MRI, a short learning curve for radiologists and registrars in our experience. The ultrasound and mammographic RL insertion device utilises an ‘unsheathing’ technique, different from other wireless devices and clips that ‘deploy forward’. The procedure needs slightly more technical awareness but is not difficult after training is completed and 1–2 procedures are performed. Mammographic localisation requires more involved technical training, but once training is complete, it is also not difficult; this is supported by minimal migration rates and lower procedure times compared to HWL. The versatility of ultrasound, mammographic, and MRI insertion affords choice of imaging modality that best shows abnormality for localisation (while considering patient comfort), providing more precision [13, 18].

Intraoperative retrieval issues occurred in 4.2% (RL) vs. 9.8% (HWL) of cases. Four out of the six RL retrieval issues at surgery were due to large fatty breasts, which may be a limiting factor, and one due to failure of detection; however, retrieval issue rates were still lower than in HWL, where retrieval issues were not related to large fatty breasts, but were due to mechanical and technical issues. SCOUT quotes a detection range of 60 mm [19], a limitation in large breasts where distance from the skin to the RL marker is larger than 60 mm, but this did not limit successful RL retrieval. Real‐time distance measurement feedback without needing calibration may also contribute to accurate device retrieval. Our results are comparable to prior literature showing successful retrieval in almost all cases [17].

### 4.2 Surgical Oncologic Outcomes

There was no statistically significant difference in margin status and breast malignancy re‐excision rates, key oncologic metrics of preoperative localisation success, between groups. Negative margin rates were similar, and re‐excision rates (a key economic outcome for hospital surgical practice) were in fact slightly less for RL (12.5%) vs. HWL (16.8%). This suggests RL is comparable in oncologic outcomes to HWL, consistent with prior literature showing similar positive margin and re‐excision rates between RL and HWL [17, 20, 21, 22]. Overall, re‐excision rates for RL are similar to rates reported in Australian literature [17, 23]. A direct comparison between these numbers to the results of this study is not accurate due to the small overall numbers in all three studies, although one can conclude that they are fairly comparable outcomes. In comparison to a pooled analysis of international studies [7], the re‐excision rates were similar (8.6%). These results overall support accurate placement and retrieval of RL, extrapolating to a similar or smaller chance of leaving margins needing re‐excision, emphasising the surgical oncologic efficacy of RL for wide local excision of impalpable breast cancers and DCIS.

### 4.3 Workflow Implications

**FIGURE 2:**
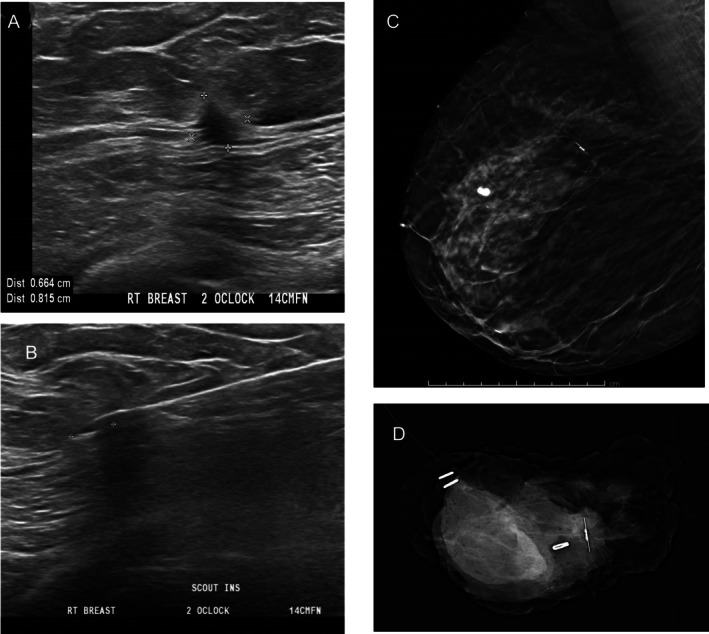
SCOUT placed into a BI‐RADS 5 impalpable spiculated mass at time of biopsy – (A) ultrasound images, (B) SCOUT insertion images, (C) subsequent post‐SCOUT insertion mammogram MLO view, and (D) wide local excision specimen X‐ray. This was an invasive ductal carcinoma, resected with clear margins.

The study results have several practical workflow implications. Firstly, RL decouples insertion from surgery, reducing same‐day scheduling pressures and theatre delays, and improving radiology and theatre scheduling, workflow and efficiency. RL also reduces patient anxiety on day of surgery, with less invasive procedures required. The aforementioned workflow benefits are common to all wireless localisation devices, not unique to RL. RL at time of biopsy was performed for unifocal impalpable BI‐RADS 5 lesions (see Figure 2 for example). This eliminates a second localisation procedure, as SCOUT serves as a ‘marker clip’ and localisation device, streamlining patient journey and reducing time to surgery. It also potentially decreases overall cost, although formal cost‐analysis studies are required to verify this. Procedure duration was favourable for RL when MRI placements were excluded (mean ~23 min RL vs. ~28 min HWL, *p* < 0.01), suggesting significant efficiency gains at the localisation step.

Similar to HWL, RL can be inserted into multiple lesions and offers ‘bracketing’ capability, where 2 reflectors are placed at boundaries of a large abnormality to define extent, enabling area to be resected en bloc. 15 mm is the minimum distance required between reflectors to prevent signal interference, with most bracketing cases encompassing a larger area.

**FIGURE 3:**
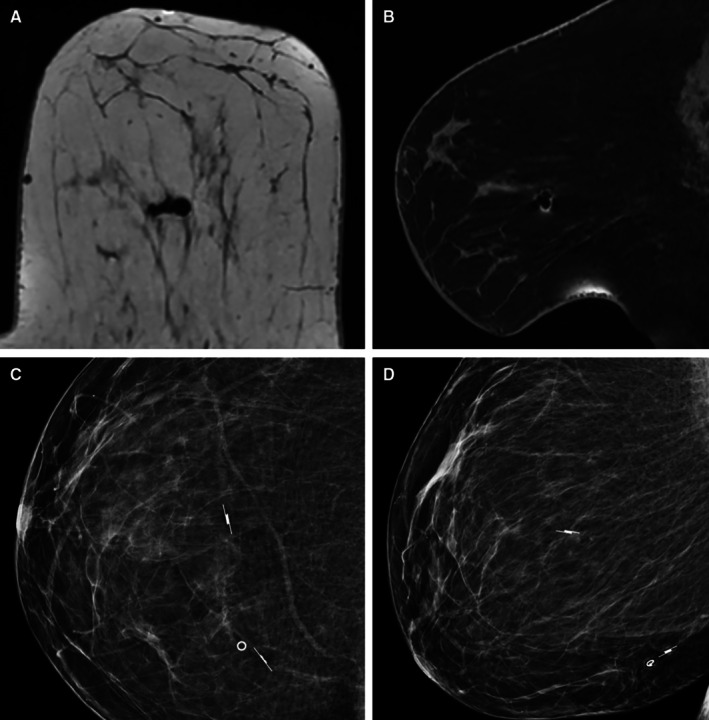
Appearance of SCOUT localiser on MRI and corresponding mammogram, inserted for non‐mass enhancement. (A) Axial 3D T1 non‐fat saturated sequence, (B) Sagittal T1 fat‐saturated non‐contrast sequence, (C) CC projection, (D) MLO projection. A second SCOUT localiser was inserted into the clipped index malignancy under ultrasound, demonstrating bracketing capability.

Unlike magnetic and radiofrequency devices, RL has minimal, clinically insignificant MRI artefact (Figure 3), enabling follow‐up diagnostic MRI imaging. This distinction is beneficial for patients undergoing preoperative or surveillance MRI, particularly when RL is performed during or prior to neoadjuvant therapy; follow‐up MRI can enable accurate surgical planning, particularly if BCS is planned. In this early experience, the MRI‐compatible insertion system provides highly precise localisation of abnormalities only seen on MRI, such as non‐mass enhancement, owing to high soft‐tissue resolution of MRI (see Figure 3). Previously, the clip placed at time of MRI biopsy was localised to assist excision of MRI abnormalities, which is less accurate, particularly if clip migration has occurred. Logistically, MRI‐guided RL is much easier than MRI‐guided HWL. Being non‐magnetic, RL also obviates the need for non‐metallic instruments in theatre.

The ability to localise axillary lymph nodes for TAD is a distinct advantage of RL over HWL. HWL is not practicable and is technically challenging for axillary nodes. This aligns with recent de‐escalation of axillary surgery [24, 25] and shift to more targeted, conservative management of the axilla, minimising morbidity of axillary dissection. The ‘mini‐SCOUT’ for axillary nodes is technically as easy to perform as breast RL in our experience.

### 4.4 Potential Limitations of SCOUT

SCOUT has several potential limitations. SCOUT is partly composed of nitinol, a nickel‐titanium alloy used in many medical devices, not suitable for patients with a severe nickel allergy. Another limitation is the inability to reposition the reflector if incorrectly placed, compared to certain hookwires which can be readjusted. However, as supported by our data, this is uncommon. SCOUT can be deactivated intraoperatively by surgical cautery, which has occurred occasionally in our cohort. Although this data was not collected, this did not preclude retrieval, as by the time the diathermy device contacts the reflector, it is visible to the surgeon and easily retrieved. Theoretically, haematoma from prior biopsy can impede the SCOUT signal; in these cases, we inserted SCOUT just anterior/superficial to the haematoma. In our study, 4 cases had a haematoma at the time of RL, and the SCOUT reflectors were successfully retrieved in all cases.

### 4.5 Cost Considerations

A perceived limitation of SCOUT is the higher cost of each individual device and surgical detection equipment ($500 AUD and ~$80,000 AUD respectively, at time of publication) compared to hookwires (~$40 AUD per wire). However, workflow advantages may offset this cost, particularly the ability to schedule theatre and radiology more efficiently due to decoupling of radiology and theatre schedules and reduction of further patient visits when RL is performed at the time of biopsy. A recent Australian surgical study supports this hypothesis [17].

### 4.6 Study Limitations

This study has important limitations. Its retrospective design and use of a historical RL and HWL cohort introduce potential selection bias and temporal confounding factors; practice patterns and case mix may have shifted during the study period. The study serves as a preliminary audit of our early RL experience after introduction.

Event counts for some outcomes were small, which limits precision and precludes robust adjusted modelling for rare events. Data completeness varied between groups for some documentation‐dependent variables; for example, haematoma was reliably recorded only for RL placements, which could bias comparative complication rates. As the study involves introduction of a new technology, radiologist and surgeon learning curves may also confound data, although this is reflective of a teaching hospital context, with new staff (especially registrars) always being trained.

We did not perform a formal health‐economic analysis or capture patient‐reported outcome measures. This would further characterise patient and system‐level impacts of replacing HWL with RL, planned in future work. Formal patient, radiologist, surgeon and paramedical (nursing, radiographer, administrative) staff satisfaction surveys could also be considered in future work.

## 5 Conclusion

RL is a safe, accurate and versatile alternative to HWL, while maintaining similar surgical oncologic outcomes. Our study results are consistent with international literature and add Australian public sector experience to the literature. SCOUT's compatibility with MRI, capacity for axillary node placement, and suitability for time‐of‐biopsy deployment, with workflow benefits of decoupling insertion and surgery, suit modern multidisciplinary breast cancer care. Findings support broader adoption in Australian breast services, with potential benefit in regional centres that lack capacity for same‐day HWL. There are also potential benefits in screening and neoadjuvant workflows, to streamline care pathways, especially for lesions suitable for time‐of‐biopsy localisation. Further prospective, multi‐centre studies, cost‐efficiency analyses, and comparative studies to other wireless technologies are needed going forward.

## Author Contributions

**Grace Carpenter:** conceptualization, writing – original draft, methodology, writing – review and editing, investigation, project administration. **Coralie Simoni:** data curation, writing – review and editing, formal analysis, investigation. **Lucy Lu:** data curation, investigation. **Jacqueline Eskander:** data curation, investigation. **Tracy Liu:** data curation, investigation. **Jennifer Yuill:** data curation, project administration. **Sophie Nightingale:** conceptualization, writing – review and editing, supervision, investigation, methodology. **Margaret Nguyen:** conceptualization, formal analysis, writing – review and editing, supervision, investigation, methodology. **Joseph Paiva:** conceptualization, formal analysis, methodology, investigation, supervision, writing – review and editing.

## Funding

The authors have nothing to report.

## Ethics Statement

Western Health Low Risk Ethics Committee (LR/25/WH/116869); consent waived for de‐identified data.

## Consent

Waiver of consent granted by the HREC for retrospective audit of de‐identified data.

## Conflicts of Interest

The authors declare no conflicts of interest.

## Data Availability

De‐identified data are available upon reasonable request and subject to institutional approvals.
